# Impaired coronary flow reserve by hyperviscosity in a mouse model of non-light chain multiple myeloma: a mechanism of coronary flow impairment at the capillary level

**DOI:** 10.1093/cvr/cvaf164

**Published:** 2025-09-16

**Authors:** Aleksandra Paterek, Filip Rolski, Mateusz Surzykiewicz, Zofia Pilch, Karol Czubak, Grażyna Hoser, Jakub Gołąb, Dominika Nowis, Tomasz Skirecki, Michał Mączewski

**Affiliations:** Department of Clinical Physiology, Centre of Postgraduate Medical Education, 99/103 Marymoncka Str., Warsaw 01-813, Poland; Department of Clinical Physiology, Centre of Postgraduate Medical Education, 99/103 Marymoncka Str., Warsaw 01-813, Poland; Department of Clinical Physiology, Centre of Postgraduate Medical Education, 99/103 Marymoncka Str., Warsaw 01-813, Poland; Department of Immunology, Medical University of Warsaw, 5 Nielubowicza Str., Warsaw 02-097, Poland; Laboratory of Experimental Medicine, Medical University of Warsaw, 5 Nielubowicza Str., Warsaw 02-097, Poland; Department of Translational Immunology and Experimental Intensive Care, Centre of Postgraduate Medical Education, 99/103 Marymoncka Str., Warsaw 01-813, Poland; Department of Immunology, Medical University of Warsaw, 5 Nielubowicza Str., Warsaw 02-097, Poland; Centre of Preclinical Research, Medical University of Warsaw, 1B Banacha Str., Warsaw 02-097, Poland; Department of Immunology, Medical University of Warsaw, 5 Nielubowicza Str., Warsaw 02-097, Poland; Laboratory of Experimental Medicine, Medical University of Warsaw, 5 Nielubowicza Str., Warsaw 02-097, Poland; Department of Translational Immunology and Experimental Intensive Care, Centre of Postgraduate Medical Education, 99/103 Marymoncka Str., Warsaw 01-813, Poland; Department of Clinical Physiology, Centre of Postgraduate Medical Education, 99/103 Marymoncka Str., Warsaw 01-813, Poland

**Keywords:** Multiple myeloma, Coronary flow, Capillaries, Blood viscosity

## Abstract

**Aims:**

Multiple myeloma (MM) is associated with cardiovascular risk, although the exact underlying mechanisms are unknown. Here, we tested the hypothesis that MM impairs coronary flow reserve (CFR) due to increased blood viscosity caused by elevated monoclonal protein concentration.

**Methods and results:**

In a mouse Vĸ*MYC model of non-light chain MM recapitulating all aspects of human disease, we showed that the disease progression was associated with progressive increase of blood and plasma viscosity. Using intravital microscopy imaging of *ex vivo* stained red blood cells, we observed reduction of CFR *in vivo* with the CFR limiting site being coronary capillaries. This was further confirmed by similar coronary flow profile in mice with hyperviscosity induced by acute hyperlipidaemia and disappearance of this MM-related CFR impairment in saline perfused *ex vivo* hearts. Of note, nitric oxide production *in vivo* was increased in the coronary circulation, especially at the capillary level, but the systemic concentration of nitric oxide metabolites was unchanged, again supporting the hypothesis that increased blood viscosity is the main culprit here. Moreover, MM progression was associated with progressive impairment of left and right ventricular function, but without histological signs of myocardial deterioration, hypertrophy, or fibrosis.

**Conclusion:**

Our study shows a potentially completely new mechanism of cardiovascular adverse effects caused by MM or more broadly by hyperviscosity syndromes, i.e. CFR impairment at the capillary level. Since capillaries, unlike larger vessels, cannot be recanalized or dilated, completely new preventive approaches are needed, such as agents affecting blood rheology.

## Graphical Abstract

**Graphical Abstract:**
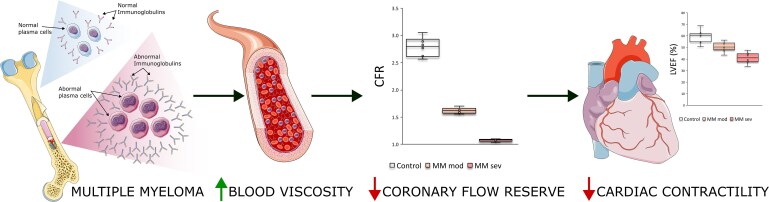

**Time of primary review: 34 days**

## 1. Introduction

Multiple myeloma (MM) is the second most common haematological malignancy in adults, characterized by clonal proliferation of malignant plasma cells in the bone marrow, monoclonal protein in the blood and urine, and end-organ damage^1^; the latter differentiates active MM from monoclonal gammopathy of undetermined significance and smoldering MM. Infection and renal failure are the main causes of early mortality. Despite significant progress in therapy, MM remains an incurable disease with a 5-year relative survival rate of 53.9%.^2^

Recently, monoclonal gammopathy of undetermined significance has been shown to be associated with increased cardiovascular (CV) morbidity, in particular of heart failure.^3^ The relationship between MM and CV morbidity and mortality is more nuanced. Older studies indicated clear association between MM and increased CV morbidity and mortality: the standardized CV mortality rate among MM patients was 1.84 times higher than in the general population.^4^ However, more recent data, from French^5^ and Korean^6^ registries, surprisingly indicate actually reduced CV events and mortality in MM patients, especially long-term survivors, but at a price of heightened major and intracranial bleeding. This could be due to initiation of intensive thromboprophylaxis in these patients.^5^ Clearly, more targeted prevention therapies are needed in MM patients to maintain efficacy, but reduce adverse effects of prophylactic management.

Light chain MM is not uncommonly associated with cardiac amyloidosis,^7^ resulting in restrictive cardiomyopathy and heart failure with preserved ejection fraction^8^ and poor prognosis, although it has also been linked with systolic dysfunction.^9^ Non-light chain MM is also associated with cardiac toxicity, but its mechanism is even less clear. Impairment of coronary flow reserve (CFR) and resulting stable coronary artery disease-like syndrome is among the possible mechanisms. Hyperviscosity related to increased serum concentrations of monoclonal protein may reduce CFR and result in cardiac dysfunction and heart failure.^10^ High output heart failure, potentially caused by arteriovenous shunts, enhanced vascularity, hyperviscosity, and increased shear stress with increased NO production have also been reported in MM patients.^11^ We have recently shown in the mouse Vĸ*MYC model of non-light chain MM recapitulating all aspects of human disease^12^ that the progression of MM is associated with increasing concentrations of plasma monoclonal protein and progressive left ventricular (LV) systolic dysfunction.^13^ However, the effects of MM on CFR have never been tested.

Experimental studies indicate that increased plasma viscosity results in increased NO synthesis^14^ through stimulation of endothelial NO synthase.^15^ On the other hand, we have shown that in the mouse model of non-light chain MM, systemic concentrations of ʟ-arginine, a precursor for NO synthesis, are progressively reduced, correlating with arginase expression in myeloid cells.^16^ So the effects of MM on endothelial function and systemic NO availability remain unknown.

Thus, the aim of this study was to investigate the effects of MM on coronary flow and endothelial function in the mouse Vĸ*MYC model of non-light chain MM.

## 2. Methods

All experiments were performed in 8–12-week-old female C57BL/6 mice obtained from the Animal House of the Medical Research Center, Polish Academy of Sciences (Warsaw, Poland). A total of 85 mice were included. Animals were housed in controlled conventional environmental conditions animal facility of the Medical Centre for Postgraduate Education in Warsaw, with water and food provided *ad libitum*. The experiments were performed in accordance with the guidelines approved by the 2nd Local Ethics Committee in Warsaw (approval nos. WAW2/003/2021 and WAW2/152/2023) and in accordance with the requirements of the EU (Directive 2010/63/EU) and Polish (Dz. U. poz. 266/15.01.2015) legislation.

### 2.1 Vκ*MYC MM model and experimental design

C57BL/6J mice were intravenously transplanted with 1 × 10^6^ Vĸ*MYC cells (a kind gift from Prof. Leif Bergsagel, Mayo Clinic College of Medicine, USA). In this syngeneic MM model, the disease develops in the spleen and in the bone marrow. Vĸ*MYC MM model tightly recapitulates human MM features, including anaemia and bone involvement^12^ as well as anti-myeloma drugs sensitivity.^17^ MM development was monitored with spleen weight and serum IgG concentration (measured with IgG Mouse ELISA Kit, ab157719, Abcam at 1–4 × 10^5^ serum dilution, according to the manufacturer’s protocol).

### 2.2 Experimental groups

**Figure 1:**
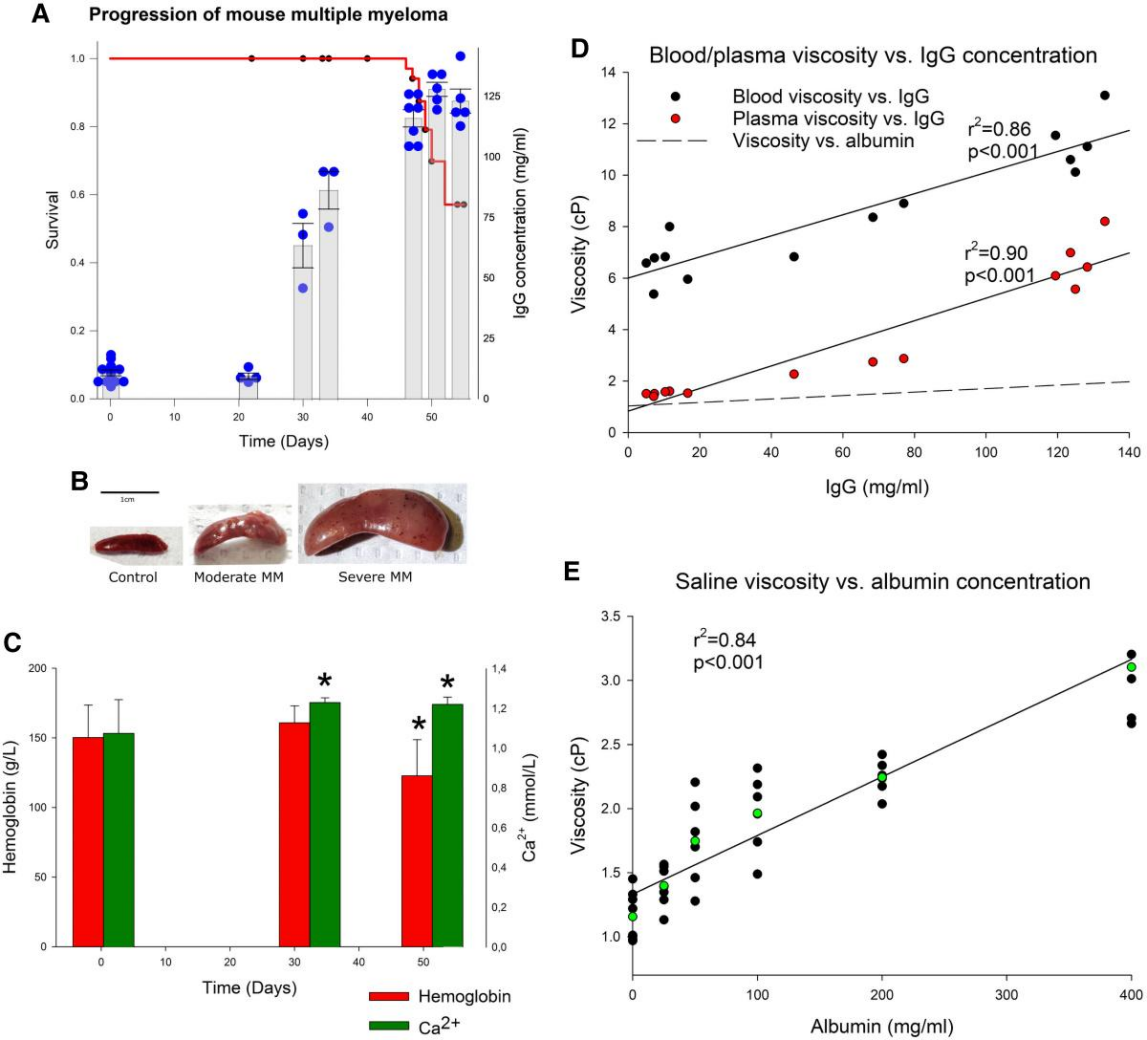
Characterization of mouse model of Vĸ*MYC MM. (*A*) Progression of MM. A solid red line indicates survival (Kaplan–Meier curve, left axis), with black dots representing censoring points (*n* = 55). Blue circles represent concentration of IgG, while bars represent mean IgG concentration ± SD at a given time point. (*B*) Representative spleen images for control, moderate MM, and severe MM corresponding to specific time points and IgG concentration in (*A*). (*C*) Mean haemoglobin (red bars) and Ca^2+^ (green bars) concentrations in control, moderate MM, and severe MM mice (*n* = 10, 15, and 17, respectively). One-way ANOVA with Tukey’s *post hoc* test. Asterisk (*) indicates *P* < 0.05 vs. control. (*D*) Correlation (Pearson) between IgG plasma concentration and blood (black circles) and plasma (red circles) viscosity with individual linear correlation lines and coefficients. Dashed line represents relation between albumin concentration in saline and viscosity of this solution, redrawn from (*E*). (*E*) Correlation (Pearson) between albumin concentration in the saline and viscosity of the solution: black circles represent individual measurements, green circles represent means for a given albumin concentration, and solid line represents the regression line with a correlation coefficient.

Control C57BL/6J mice (*n* = 27), mice with moderate MM (*n* = 20, serum IgG > 20 and <100 mg/mL), and severe MM (*n* = 35, serum IgG > 100 mg/mL) were used. Inoculation was unsuccessful in three mice. Spontaneous mortality occurred only in severe MM group (*n* = 12, see *Figure 1*), so eventually 23 mice from this group were available for testing. Fourteen control mice (nine control and five control + Clinoleic infusion), and nine moderate MM and nine severe MM mice underwent intravital microscopy imaging; intraprocedural mortality was three, three, and three animals in control, moderate MM, and severe MM, respectively, due to bleeding, accidental extubation or suction-induced ischaemia. Separate subgroups of mice (each *n* = 6) were used for histology to avoid tissue contamination with agents given during intravital imaging. Eventually, separate subgroups of mice (seven, five, and eight animals from control, moderate MM, and severe MM groups, respectively) were used for experiments to obtain invasive measurements of blood pressure and cardiac function.

### 2.3 Echocardiography imaging and invasive catheterization

Transthoracic echocardiography was performed using E-cube 15 Platinum (Alpinion Medical Systems) with 17 MHz linear transducer with mice being lightly sedated by inhaled isoflurane (approximately 1%) to maintain the heart rate > 400 b.p.m. After sedation, mice were placed on the heating pad to sustain proper body temperature. Images of the parasternal short-axis view at the papillary muscle level, parasternal long-axis view, and the apical four-chamber view were recorded. LV end-diastolic (LVEDV) and end-systolic (LVESV) volumes were determined from the parasternal long-axis view. LV ejection fraction, a marker of LV systolic function, was calculated from Simpson method. LV diastolic function was assessed by pulsed Doppler of the mitral inflow, with use of E wave to A wave ratio (E/A), isovolumic relaxation time (IVRT), and ejection time (ET). Isovolumic contraction time (IVCT) served as an additional parameter of LV contractility. Pulmonary acceleration time (PAT) and PAT/PET (pulmonary ET) ratio were obtained from analysis of pulsed Doppler pulmonary artery flow. Tricuspid annular plane systolic excursion (TAPSE) and mitral annular plane systolic excursion (MAPSE) were obtained from four-chamber view. All measurements were obtained by one observer blinded to the study groups.

A subgroup of control, moderate, and severe MM mice was anaesthetized with inhaled 2–2.5% isoflurane and intubated. The chest was subsequently opened and the LV apex was exposed, punctured with a 25G needle, and a microtip pressure-volume (PV) catheter (SPR-838, Millar Instruments; Houston, TX) was inserted into the LV. Then, anaesthesia was reduced to 1% isoflurane to maintain heart rate > 400 b.p.m. and LV pressure, maximal slope of systolic pressure increment (+dP/dt *max*) and diastolic pressure decrement (−dP/dt *max*), and systolic and diastolic blood pressure were obtained. Subsequently, the tip of the catheter was inserted to the aorta to obtain aortic pressures.

### 2.4 Measurements of viscosity and blood analyses

Blood and plasma viscosity were measured with a microviscometer microVISC (Rheosense, CA, USA) at a shear rate of 100 s^−1^ and at 37°C. After calibration with pure water, approximately 10 µm of blood or plasma was injected and analysed in triplicates. Blood serum analyses were performed using ABL90 Flex Plus (Radiometer, Denmark) analyzer. Nitrite concentration in the blood plasma was determined by a gas phase chemiluminescence reaction of NO with ozone using a Nitric Oxide Analyzer (NOA, Sievers Instruments). In this method, nitrate is reduced to NO gas in the purge vessels of the analyser by potassium iodide in glacial acetic acid.^18^ Blood (100 µL) was collected from the tail vein.

### 2.5 Intravital microscopy imaging

Mice were anaesthetized with ketamine and xylazine (100 and 5 mg/kg body weight, respectively, intraperitoneal). The trachea was exposed microsurgically, and animals were put on a ventilator and ventilated with a tidal volume of 250 µL. The chest was opened, and the heart was exposed; subsequently, the mouse was inoculated into the LV with a 5 µg of Alexa Fluor® 647 anti-mouse CD31 Antibody (BioLegend, cat. no. 102416) to outline the contours of blood vessels (see Supplementary material online, *Movies S1*–*S3*) and mouse red blood cells (RBCs) stained *ex vivo* with a lipophilic tracer 1,1′-dioctadecyl-3,3,3′,3′-tetramethylindocarbocyanine perchlorate (Sigma, cat. no. 42364) (an equivalent of 10 µL of whole blood) to visualize blood flow. A suction cardiac camera (with suction pressure of approximately 80 mmHg) was used to provide stabilization of a beating heart and live imaging of epicardial coronary vessels was performed with an intravital microscope (IVM-CMS3, IVIM Technology, Seoul, Korea). Magnification ×20 was used. The conventional field of vision was 500 µm × 500 µm, and the depth of the signal was 20 µm. After confirmation of positive anti-CD31 staining (visibility of cardiac capillaries) and adequate perfusion (stained RBCs flowing through the capillaries) as well as cardiac function (heart rate > 400 b.p.m.), baseline RBC flow velocity through capillaries, arterioles (<50 µm in diameter), and venules (<50 µm in diameter) as well as arteriolar and venular diameter were recorded (see Supplementary material online, *Movies S1*–*S3*). Subsequently, adenosine (Sigma, 30 µL bolus, 250 µg in phosphate buffered saline) was injected into the LV and the measurements of RBC flow velocity and vessel diameters were repeated to obtain both CFR. CFR was defined as an average capillary RBC flow velocity after adenosine/baseline capillary RBC flow velocity. Our preliminary results indicated that maximum capillary RBC flow velocity occurs 1–2 min after adenosine injection. The frame rate of the recorded movies was 33 images per second.

RBC flow velocity was counted manually post imaging. The sequences of at least four consecutive images (each taken 0.03 s after the previous one) with the same RBC visible in the same blood vessel (capillary, arteriole, or venule) were identified and superimposed so that the distance between start position and end position could be measured. Eventually, the distance was divided by the time that elapsed between the first and the last image of the series, giving an average RBC flow velocity. At least 10 RBCs were analysed for each blood vessel type and for each field of vision; data from at least two fields of vision were combined giving an average RBC flow velocity for each vessel type for each animal. The same strategy was repeated for images obtained after adenosine injection.

To increase blood viscosity in otherwise healthy animals, after the above mentioned procedure, three mice were given an intravenous lipid emulsion. Clinoleic (Omegeaven, refined olive oil and refined soybean oil 20% lipid emulsion, 3 µL/g of body weight) was given as a bolus injection into the LV. Two minutes later, the above mentioned procedure of CF measurement was repeated, including adenosine administration.

The imaging was performed for maximum 60 min. Capillaries were counted in at least three different LV sites and their number averaged. The final result was expressed as the number of capillaries (estimated in long-axis view) per 500 µm (determined by the depth of the signal penetration, corresponding to a cross-section area of 10 mm^2^). Capillary patency was expressed as the ratio of capillaries with at least one RBC flowing per 20 s of observation. Mice body temperature was monitored and maintained at 37°C during the procedure.

At the conclusion of the experiment, the ketamine/xylazine overdose was given and diaminofluorescein-2 diacetate (DAF2-DA), a cell-permeable nitric oxide (NO) probe that is hydrolysed to DAF2 by intracellular esterases, was injected into LV to visualize NO production (24 µmol in 0.5 mL PBS). Imaging was performed immediately at three different areas of LV. In selected experiments *N*(gamma)-nitro-L-arginine methyl ester (L-NAME, Merck, Germany), 50 mg/kg dissolved in 1 mL PBS was injected over 5 min into LV prior to DAF2-DA staining. NO production was measured as mean fluorescence intensity in capillaries, small vessels (<50 µm diameter), and intermediate vessels (>50 µm diameter) at depths 20–30 µm to minimalize signal loss associated with the depth of imaging. Image analysis was performed using ImageJ software (Version 2.14, NIH, USA).

In four mice, imaging of blood flow through the liver and the small intestine was performed. Both these organs were imaged using simple organ immobilization and a non-suction camera.

In six mice, the imaging was unsuccessful (probably due to myocardial ischaemia caused by the CF blockade due to applied suction, manifested as lack of RBC flowing through the coronary vessels).

Ketamine and xylazine overdose (200 and 10 mg/kg body weight, respectively, intraperitoneal) was used for euthanasia.

### 2.6 Langendorff-perfused hearts

Three hearts from each of the control and MM severe groups were extracted and Langendorff-perfused with Krebs–Henseleit buffer using an isolated rat lung perfusion system (Hugo Sachs Elektronik Harvard Apparatus, March Hugstetten, Germany), as described previously.^13^ After 10 min of perfusion and coronary flow stabilization, the perfusion pressure was varied (40, 60, and 80 mmHg) and coronary flow was recorded using an electromagnetic flowmeter to plot CF against coronary perfusion pressure and a curve reflecting *ex vivo* pulmonary vascular resistance was generated.

### 2.7 Histology

Hearts were collected and fixed in 10% neutral-buffered formalin for 24 h at room temperature and subsequently embedded in paraffin. Paraffin blocks were cut in transversal sections of 5 µm and stained with H&E and Masson’s trichrome. The stained sections were examined using an Olympus microscope (Olympus Corporation, Tokyo, Japan) and photographed. H&E staining was used to determine the basic tissue morphology and measure of wall thickness. LV, right ventricular (RV), and septum thickness were calculated in ImageJ by measuring wall thickness in 5–14 different areas.

Fibrosis was analysed using the Masson’s trichrome kit according to manufacturer’s instructions (Biognost). Quantification of interstitial fibrosis was calculated using ImageJ software and Colour Deconvolution 2 plugin. Interstitial fibrosis was calculated separately in LV and RV: percentage of fibrosis was calculated in 3–9 random regions of interest per image in 4–10 images per heart. Perivascular fibrosis was quantified as previously described.^19^ In brief, trichrome-stained images of intramyocardial coronary blood vessels, ranging from 20 to 250 μm in diameter, were captured. Blue fibrillar collagen staining identified adventitial (perivascular) fibrosis, while red staining marked the blood vessel smooth muscle (tunica media). The area of adventitial fibrosis was determined by subtracting the area of the blood vessel (lumen + smooth muscle) from the combined area of the adventitia and blood vessel, using ImageJ. To account for differences in the vessel size, the ratio of the adventitial area to the vessel area was calculated, representing the normalized perivascular fibrotic area. For each heart, measurements were taken from three to seven intramyocardial coronary arteries.

For wheat germ agglutinin (WGA) staining, cardiac paraffin sections (5 μm) were kept at 60°C overnight and then deparaffinized in xylene for 3 × 5 min and rehydrated in 100% ethanol for 2 × 5 min and 96% ethanol for 2 × 5 min. After incubation with antigen retrieval solution (citrate buffer solution, pH 6.0) at 99°C for 20 min, the slides were washed with double-distilled water and Tris-buffered saline with 0.1% Tween® 20 Detergent (TBST). After washing, the sections were incubated with WGA Alexa Fluor 594 conjugate (1:200; Invitrogen, cat. no. W11261) for 10 min at room temperature. The slides were covered by coverslip using Fluorescent Mounting Medium (ROTI®Mount FluorCare, cat. no. HP19.1).

Quantification of cardiomyocyte size in long and short axis was done using ImageJ in a blinded fashion. Only cardiomyocytes with a fully intact cell membrane had their boundaries traced and cross-sectional area and dimensions determined.

### 2.8 Statistical analysis

Data are shown as means ± standard deviation (SD) or median ± interquartile ranges, depending on distribution. Sigma Plot 15.0 (Inpixon, CA, USA) was used for statistical analyses. The normality of data distribution was tested using Shapiro–Wilk test. For statistical analyses of two groups, unpaired two-tailed *t*-test was used. For statistical analyses of three or more groups, one-way or two-way analysis of variance (ANOVA) was used followed by *post hoc* Tukey’s or Dunnett’s multiple comparisons tests. A *P*-value of less than 0.05 was considered statistically significant. The survival rate was computed using Kaplan–Meier plots and analysed with log-rank test. Information on the group size, statistical analysis used, and *P*-values is provided in the figure captions.

## 3. Results

### 3.1 Characterization of Vκ*MYC MM model of MM

We used a murine MM model, syngeneic with C57BL/6 mice. Mice were inoculated intravenously with cells isolated from the spleens of diseased transgenic Vκ*MYC mice.^17^ There was a progressive increase in the serum immunoglobulin G (IgG) levels starting from Week 4 after the inoculation (*Figure 1A*), followed by mortality starting from Day 45 (more than 40% of the animals were dead by the end Week 8 after inoculation of Vκ*MYC cells) (*Figure 1A*). In three cases, inoculation was unsuccessful. In our study, we used two groups of mice, those with moderate and severe MM, corresponding to Weeks 4–5 and 7–8 after inoculation of tumour cells, respectively. The serum IgG concentrations in these groups were 30–90 and >100 mg/mL (*Figure 1A*).

The progression of MM was accompanied by the increase in spleen weight (up to 27-fold in severe MM group; *Figure 1B* and *Table 1*), mild reduction of haemoglobin (*Figure 1C*), and haematocrit (*Table 1*). A significant increase in the plasma Ca^2+^ was observed in mice with MM (*Figure 1C*), although there was no increase in the parameters that might indicate renal dysfunction, such as serum creatinine or urea (*Table 1*), nor histological evidence of renal involvement (not shown) or changes in renal weight (*Table 1*). Altogether, these parameters reflected the well-known course of the human disease with anaemia and hypercalcaemia.

**Table 1:** Morphometric and blood serum analyses in control and MM mice

	Control (*n* = 27)		Moderate MM (*n* = 13)		Severe MM (*n* = 25)	
	Mean	SD	Mean	SD	Mean	SD
Body weight, g	23.25	3.18	22.33	1.40	25.62	2.62
Spleen, g	0.076	0.029	**0.291** ^a^	0.095	**2**.**081**^a^	0.709
Heart, g	0.139	0.042	0.127	0.011	0.154	0.020
Kidney, g	1.39	0.15	1.49	0.24	1.45	0.24
Haemoglobin, g/L	150.3	23.3	160.8	12.3	**122**.**8**^a^	26.0
Haematocrit, %	46.0	7.1	49.3	3.7	**37**.**6**^a^	8.0
pH	7.19	0.04	7.31	0.08	7.27	0.05
pCO_2_, mmHg	53.6	11.4	43.9	9.8	46.4	9.5
pO_2_, mmHg	54.3	4.7	59.2	11.0	**48**.**6**^a^	6.7
sO_2_, %	70.5	4.8	74.6	7.5	**54**.**1**^a^	10.0
Creatinine, µmol/L	29.4	2.3	27.1	3.2	29.5	5.3
Urea, mmol/L	9.75	0.83	8.85	1.44	9.84	1.78
Glucose, mg/dL	161	27	183	16	146	18
Lactate, mmol/L	3.55	2.57	4.73	1.01	5.15	0.96
K^+^, mmol/L	6.3	1.2	6.1	0.7	7.2	0.8
Na^+^, mmol/L	150	2	152	1	154	3
Ca^2+^, mmol/L	1.08	0.17	**1**.**23**^a^	0.02	**1**.**22**^a^	0.04
Cl^−^, mmol/L	117	5	118	1	117	4
Osmolarity, mOsm	323	1	324	1	325	5
Anion gap, c	11	1	12	1	14	2

^a^Bold numbers indicate statistically significant (*P* < 0.05) differences vs. control. One-way ANOVA with Tukey’s *post hoc* test.

### 3.2 Blood and plasma viscosity

In control animals, plasma and blood viscosity were 1.52 ± 0.07 cP and 6.59 ± 0.89 cP, respectively. The values increased to 2.62 ± 0.32 cP and 8.03 ± 1.07 cP in moderate MM and to 6.65 ± 1.00 cP and 11.30 ± 1.42 cP in severe MM, respectively. Thus, plasma viscosity was quadrupled, while blood viscosity was doubled in severe MM vs. control mice. Both blood (*r*^2^ = 0.86) and plasma (*r*^2^ = 0.90) viscosity exhibited significant correlation with plasma IgG concentrations (*Figure 1D*). Of note, the correlation between viscosity and concentration of albumin dissolved in saline was also linear (*Figure 1E*), but the slope of the regression was almost 10-fold steeper for MM IgG protein in plasma or blood than for albumin in saline (indicated by dashed line in *Figure 1D* for the sake of comparison), indicating that MM IgGs generate much higher viscosity than the same concentration of albumin.

### 3.3 Coronary blood flow and coronary reserve

**Figure 2:**
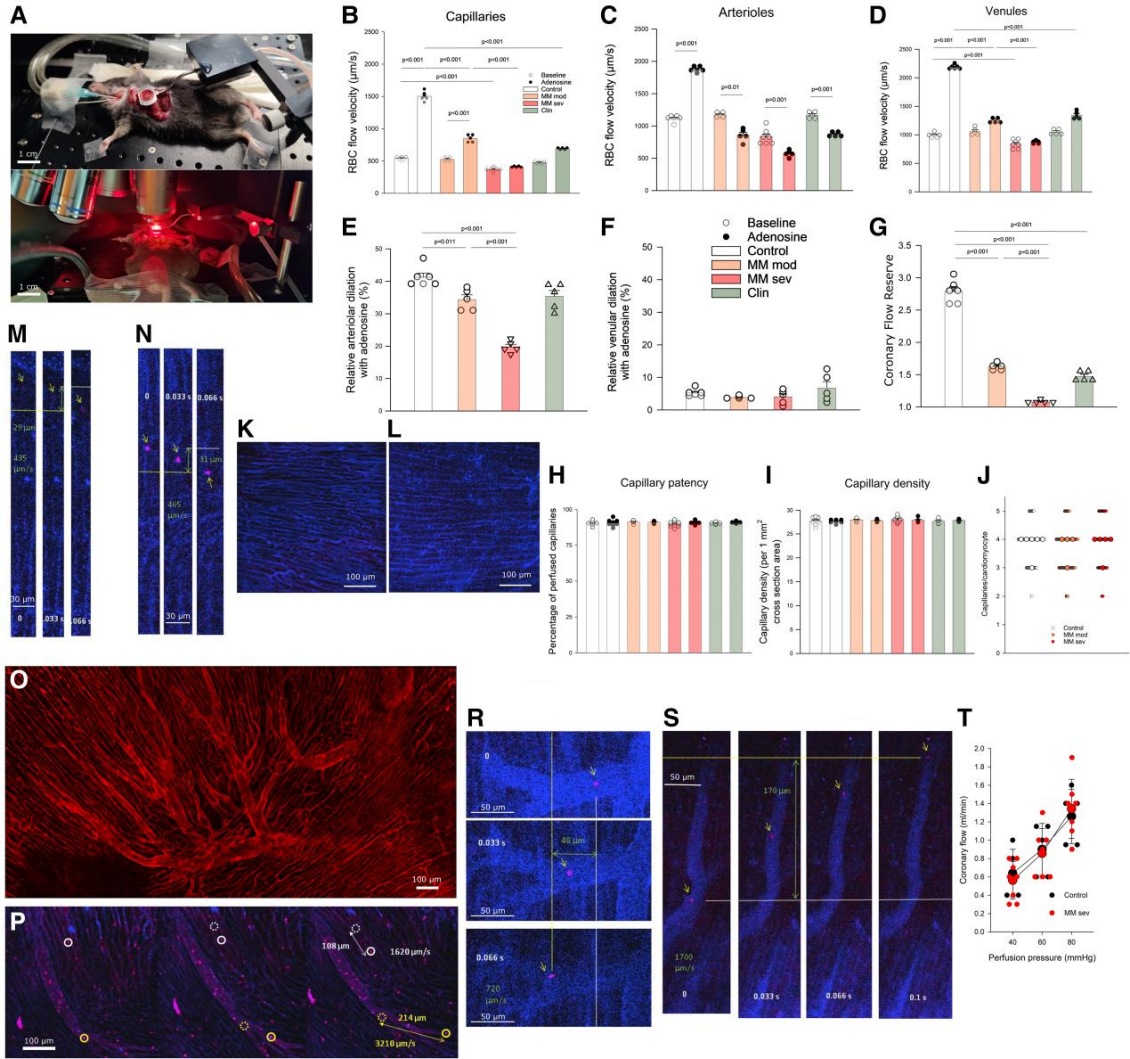
Coronary flow and CFR in Vĸ*MYC MM and control mice. (*A*) An anaesthetized and intubated mouse with opened chest and exposed heart and a cardiac imaging chamber suctioned to the beating left ventricle, ready for imaging (upper panel). A mouse during cardiac imaging using an intravital microscope (lower panel). (*B–D*) RBC flow velocity through subepicardial coronary capillaries (*B*), small arterioles (<50 µm in diameter) (*C*), and small venules (<50 µm in diameter) (*D*) in control (*n* = 7), moderate MM (*n* = 5), and severe MM (*n* = 6) mice and in control mice injected with a lipid emulsion (Clinoleic, CLIN, *n* = 5), at baseline and following injection of a bolus of adenosine (ADO) into the LV chamber. Individual dots (white—baseline, black—after adenosine) represent means for a single animal calculated from at least 10 measurements. Bars represent means ± SDs. Two-way ANOVA with Tukey’s *post hoc* test was used to calculate significance. (*E* and *F*) Maximum relative arteriolar (baseline diameter < 50 µm) (*E*) and venular (baseline diameter < 50 µm) (*F*) dilation within 2 min of injection of ADO bolus. Individual dots represent means for a single animal calculated from at least 10 measurements. Bars represent means ± SDs. One-way ANOVA with Tukey’s *post hoc* test. (*G*) CFR calculated as maximum to baseline capillary RBC velocity. Individual dots represent means for a single animal calculated from at least 10 measurements. Bars represent means ± SDs. One-way ANOVA with Tukey’s *post hoc* test. (*H*) Capillary patency and (*I*) capillary density in control, moderate MM, and severe MM mice and in control mice injected with a lipid emulsion (Clinoleic, CLIN) at baseline and following injection of a bolus of adenosine (ADO). Individual dots represent means for a single animal calculated from at least 10 measurements. Bars represent means ± SDs. Two-way ANOVA with Tukey’s *post hoc* test. (*J*) Number of capillaries supplying a single cardiomyocyte based on WGA staining of control, moderate MM, and severe MM hearts. Large circles represent medians for individual animals; small circles represent individual measurements. One-way ANOVA on ranks. (*K* and *L*) Representative images of subepicardial coronary capillaries stained with anti-CD31 (blue), in control (*K*) and severe MM (*L*) hearts. (*M* and *N*) A representative example of calculation of RBC flow velocity in coronary capillaries. Three consecutive images obtained at 0.033 s interval (frame rate 30 frames per second). (*O*) Panoramic view of subepicardial coronary arterial tree and capillaries (stained with anti-CD31), demonstrating regular branching pattern and parallel course of coronary capillaries to long axes of cardiomyocytes. (*P–S*) Representative examples of measurement of RBC velocity in coronary capillaries and arteriole (*P*) and arteriole (*S*) following ADO injection and at baseline (*R*). (*T*) Perfusion pressure—coronary flow relation obtained in isolated, Langendorff-perfused control and severe MM hearts, perfused with saline. Small dots represent individual hearts; large dots represent means ± SD for a given group and perfusion pressure.

**Figure 3:**
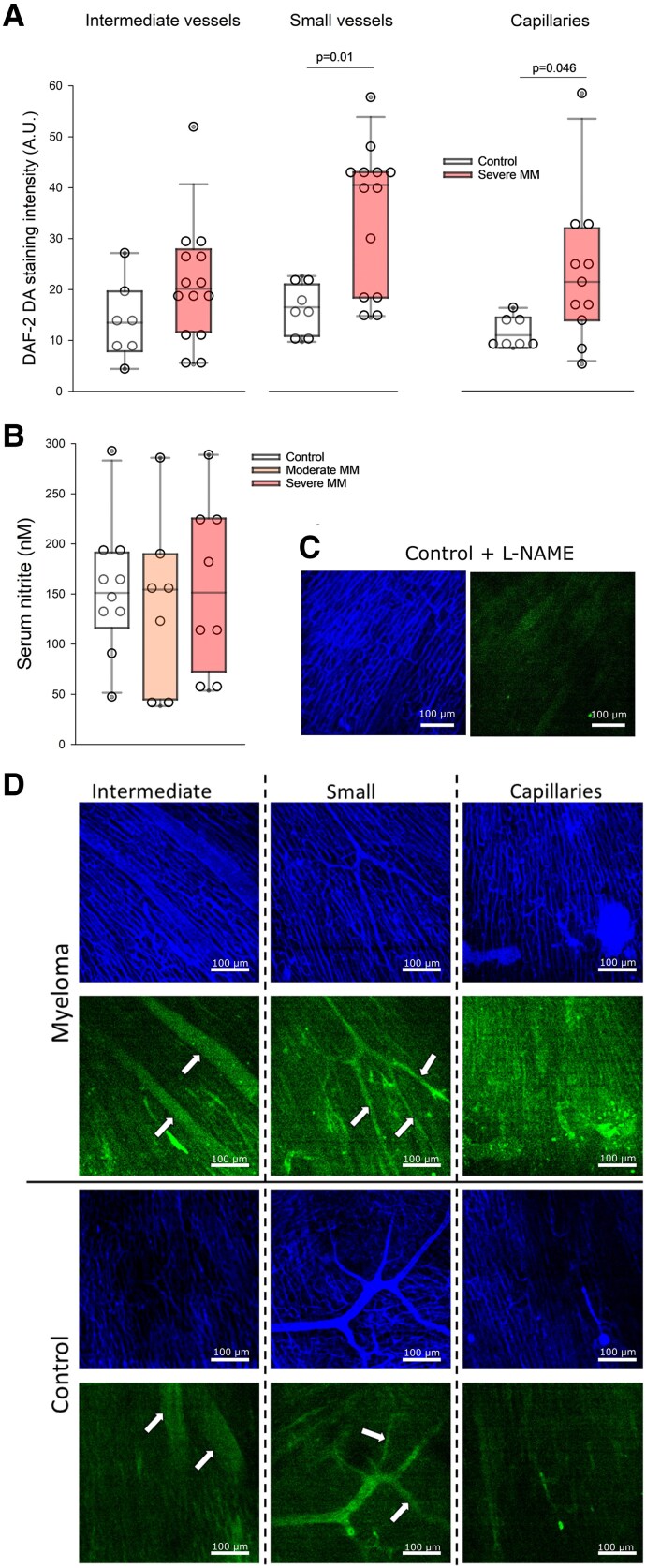
Coronary endothelial function and NO availability in Vĸ*MYC MM and control mice. (*A*) NO endothelial availability in subepicardial coronary intermediate blood vessels (arterioles and venules 50–100 µm in diameter), small blood vessels (10–50 µm in diameter), and capillaries, in control and severe MM mice. Circles represent means for individual animals; box plot represents median and interquartile ranges + maximum/minimum. Mann–Whitney rank sum test. (*B*) Serum nitrite, a measure of systemic NO production, in control, moderate MM, and severe MM mouse. Circles represent means for individual animals; box plot represents median and interquartile ranges + maximum/minimum. ANOVA on ranks. (*C* and *D*) Representative images of small, intermediate coronary blood vessels and capillaries. Blue, anti-CD31 staining of coronary endothelium; green, DAF-2 signal proportional to endothelial NO availability. White arrows indicate individual blood vessels.

Coronary blood flow was assessed using single RBC tracking with an intravital microscope (*Figure 2A*; Supplementary material online, *Movies S1*–*S5*). An average resting RBC flow velocity in LV epicardial capillaries was 540 ± 28 µm/s (*Figure 2B*). The distribution of individual capillary RBC flow velocities was quite narrow (see Supplementary material online, *Figure S1A–C*). An average RBC flow velocity was twice as high in small coronary arterioles (average diameter 29 ± 3 µm), 1117 ± 50 µm/s (*Figure 2C*), and small coronary venules (average diameter 30 ± 3 µm), 992 ± 43 µm/s (*Figure 2D*). Injection of a bolus of adenosine into the LV induced rapid dilation of small coronary arterioles (by 41 ± 3%) within 1 min (*Figure 2E*), no change of diameter of coronary venules (*Figure 2F*), reduction of coronary resistance, and increase in average capillary RBC velocity (1504 ± 90 µm/s, *Figure 2B*). This corresponded to almost three-fold increase of coronary flow, representing three-fold CFR (*Figure 3G*), since the capillary patency did not change (*Figure 2H*). Of note, an average arteriolar RBC flow velocity increased only by 68% with adenosine (*Figure 2C*) due to concurrent arteriolar dilation. At the same time, an average venular RBC flow velocity increase was over 120% (*Figure 2D*).

In mice with moderate MM, an average resting capillary RBC velocity was unchanged but maximum velocity was reduced by 44% (*Figure 2B*), corresponding to CFR reduction from 2.8 to 1.6 (*Figure 2G*). Because neither capillary density (*Figure 2I–L*) nor capillary patency (*Figure 2H*) changed in either moderate or severe MM, this reduction of RBC velocity, at baseline and after adenosine, reflected true volumetric CF and CFR, respectively. Moreover, lack of changes of capillary density and patency after adenosine injection either in control or in MM mice (*Figure 2H* and *I*) argues against capillary recruitment as a mechanism of coronary flow increase.

Since arteriolar dilation was only mildly impaired, arteriolar RBC velocity actually decreased, strongly pointing to coronary capillaries as the source of limitation of CFR. Since venules did not dilate, the RBC velocity tended to increase there, mirroring increased capillary flow. In mice with severe MM, an average resting capillary RBC velocity was reduced by 31% vs. controls, while coronary flow increase after adenosine was in fact abolished, indicating reduction of resting CF and no CFR (*Figure 2B* and *G*). The arteriolar dilation was halved (*Figure 2E*), but again in face of unchanged capillary RBC velocity after adenosine injection (*Figure 2B*) led to a significant reduction of RBC flow velocity through coronary arterioles, further supporting the observation that capillaries limit both resting CF and CRF in this group.

Lack of capillary and venular RBC velocity increase despite arteriolar dilation in moderate and severe MM was highly indicative of increased capillary resistance being a limiting factor for CFR and CF. To further support this hypothesis, we infused a lipid emulsion, Clinoleic, that acutely increases blood viscosity in healthy mice. Indeed, we found similar results as with MM: reduced CF reserve (reduced capillary RBC velocity increase with adenosine; *Figure 2B*) in spite of preserved arteriolar dilation (*Figure 2E*). These effects seemed to exhibit dose response relation, since higher Clinoleic dose that increased blood viscosity even further tended to affect CFR even more (see Supplementary material online, *Figure S1D–F*). *Figure 2M–S* present individual measurements of RBC flow velocity through various coronary vessels and the epicardial coronary arterial tree and capillaries, demonstrating regular branching pattern and parallel course of coronary capillaries to long axes of cardiomyocytes.

To further verify the hypothesis that increased blood viscosity related to MM IgG protein limits CF and CFR in MM mice, we obtained pressure-flow relations in Langendorff-perfused hearts, reflecting coronary resistance in hearts perfused with saline offering 10-fold lower resistance than blood, to see if coronary vessel related factors rather than blood related factors could affect coronary resistance in MM hearts. As shown in *Figure 2T*, pressure-flow relation profiles reflecting coronary resistance were superimposable in control and severe MM hearts, again supporting the observation that blood-related factors are responsible for abnormal CF pattern in this mouse MM model.

Finally, we imaged resting RBC flow velocity through hepatic and small intestinal blood vessels (see Supplementary material online, *Figure S2*). In neither of these vascular beds, capillary blood flow was impaired in severe MM, indicating that coronary flow is specifically affected by MM.

### 3.4 Coronary endothelial function and NO availability

In normal hearts, the greatest NO production, assessed in vivo using DAF-2 DA vascular staining, was in blood vessels (mixed arteries and veins) of intermediate (50–100 µm in diameter) and small (10–50 µm in diameter) size, while capillaries produced weaker NO signal (*Figure 3A*). Severe MM resulted in a significant increase in NO production in coronary capillaries and small vessels (by 100%), but not in larger vessels. Serum nitrite concentration, reflecting global endothelial NO production, did not change either in moderate nor severe MM (*Figure 3B*). *Figure 3C* verifies that DAF-2 DA signal is reduced by more than 90% after administration of an inhibitor of NO production, N(gamma)-nitro-L-arginine methyl ester (L-NAME), confirming that it reflects predominantly NO production, while *Figure 3D* presents individual images of DAF-2 DA stained blood vessels in control and severe MM hearts.

### 3.5 Cardiac function

**Figure 4:**
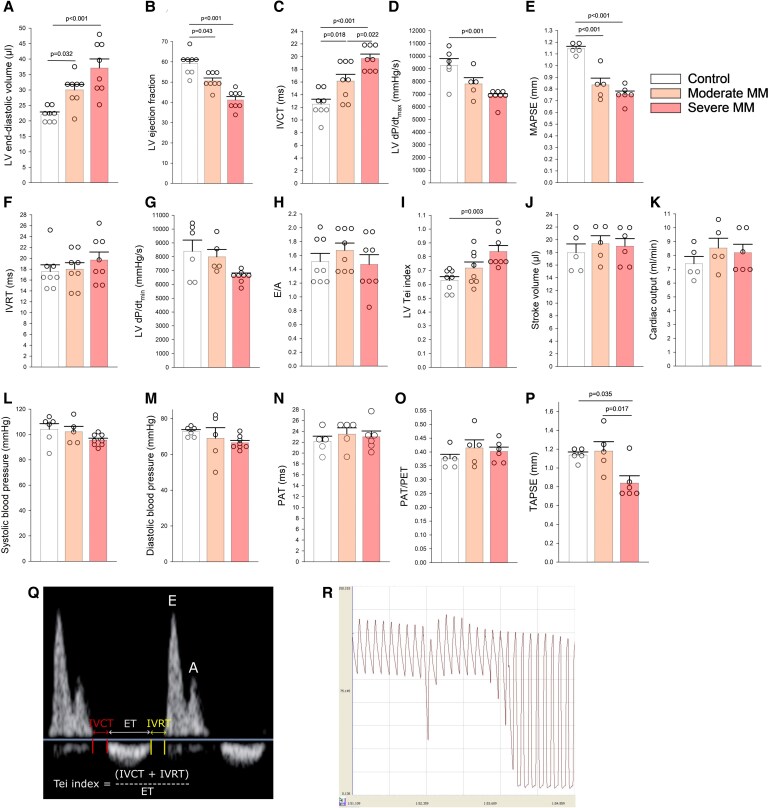
Cardiac function and structure in Vĸ*MYC MM and control mice. (*A–R*) Echocardiographic and invasive catheterization parameters of LV and RV function and aortic blood pressure in control, moderate MM, and severe MM mice. (*A*) LV diastolic volume, a marker of LV dilation, (*B*) LV ejection fraction, and (*C*) IVCT as well as (*D*) maximal slope of diastolic pressure decrement (−dP/dt *max*) and (*E*) MAPSE, markers of LV systolic function. Markers of LV diastolic function: (*F*) IVRT and (*G*) maximal slope of diastolic pressure decrement (−dP/dt *max*), reflecting active relaxation, and (*H*) E wave to A wave (E/A) ratio, reflecting passive diastolic compliance. (*I*) LV Tei index [its formula shown on (*Q*)], reflecting combined systolic and diastolic LV function. (*J*) Stroke volume and (*K*) cardiac output, markers of pumping performance of the heart. (*L*) Systolic and (*M*) diastolic aortic pressure derived from invasive catheterization. (*N*) Pulmonary artery acceleration time (PAT), a marker of pulmonary vascular resistance. (*O*) PAT/pulmonary artery ET (PAT/PET), a marker of pulmonary vascular resistance normalized to heart rate. (*P*) TAPSE, a marker of longitudinal RV contraction, reflecting RV systolic function. For (*A–P*): Individual dots represent values for a single animal, and bars represent means ± SDs. One-way ANOVA with Tukey’s *post hoc* test. (*Q*) An image of Doppler transmitral flow with marked IVCT, IVRT, and ET and formula for calculation of Tei index. (*R*) An invasive recording of LV and aortic blood pressure with a drop of diastolic pressure indicating the crossing of the aortic valve by the catheter that was slowly pulled back from the aorta to the LV. Vertical axis: pressure in mmHg; horizontal axis: time in seconds.

To gain insight into potential consequences of abnormalities in CF and CFR related to MM, we first assessed cardiac function using echocardiography and invasive catheterization of the LV and aorta. As *Figure 4* demonstrates, indices of LV dilation (*Figure 4A*) and systolic function: ejection fraction, IVCT, maximal slope of systolic pressure increment (+dP/dt *max*), and mitral annulus posterior systolic excursion (MAPSE) (*Figure 4B–E*) were already impaired in moderate MM and abnormalities worsened with the progression of the disease. However, LV diastolic function [IVRT and maximal slope of diastolic pressure decrement (−dP/dt *max*), reflecting active relaxation and E/A, reflecting passive LV compliance] was unaffected (*Figure 4F–H*). LV Tei index, a measure of combined systolic and diastolic function, was mildly reduced only in severe MM, and this impairment was driven by reduction of systolic function (*Figure 4I*). Moreover, overall LV pumping function, as reflected by LV systolic volume and cardiac output (*Figure 4J* and *K*), was also preserved. Neither systolic nor diastolic aortic pressure was affected by MM (*Figure 4L* and *M*). Indices of pulmonary vascular resistance and pulmonary artery pressure, PAT and PAT to pulmonary ET (PET) ratio, were not affected (*Figure 4N* and *O*); however, tricuspid annulus posterior systolic excursion (TAPSE) was impaired, though only in severe MM (*Figure 4P*). Thus, echocardiography provided the image of a selective LV systolic dysfunction accompanied by impairment of RV systolic function and preserved global cardiac pumping ability and systemic blood pressure. Of note, abnormalities of LV function appeared earlier in the course of the disease and were more severe than that of RV function. *Figure 4Q* presents an image of Doppler transmitral flow with presentation of various indices derived from this imaging, while *Figure 4R* presents an invasive measurement of LV and aortic blood pressure.

**Figure 5:**
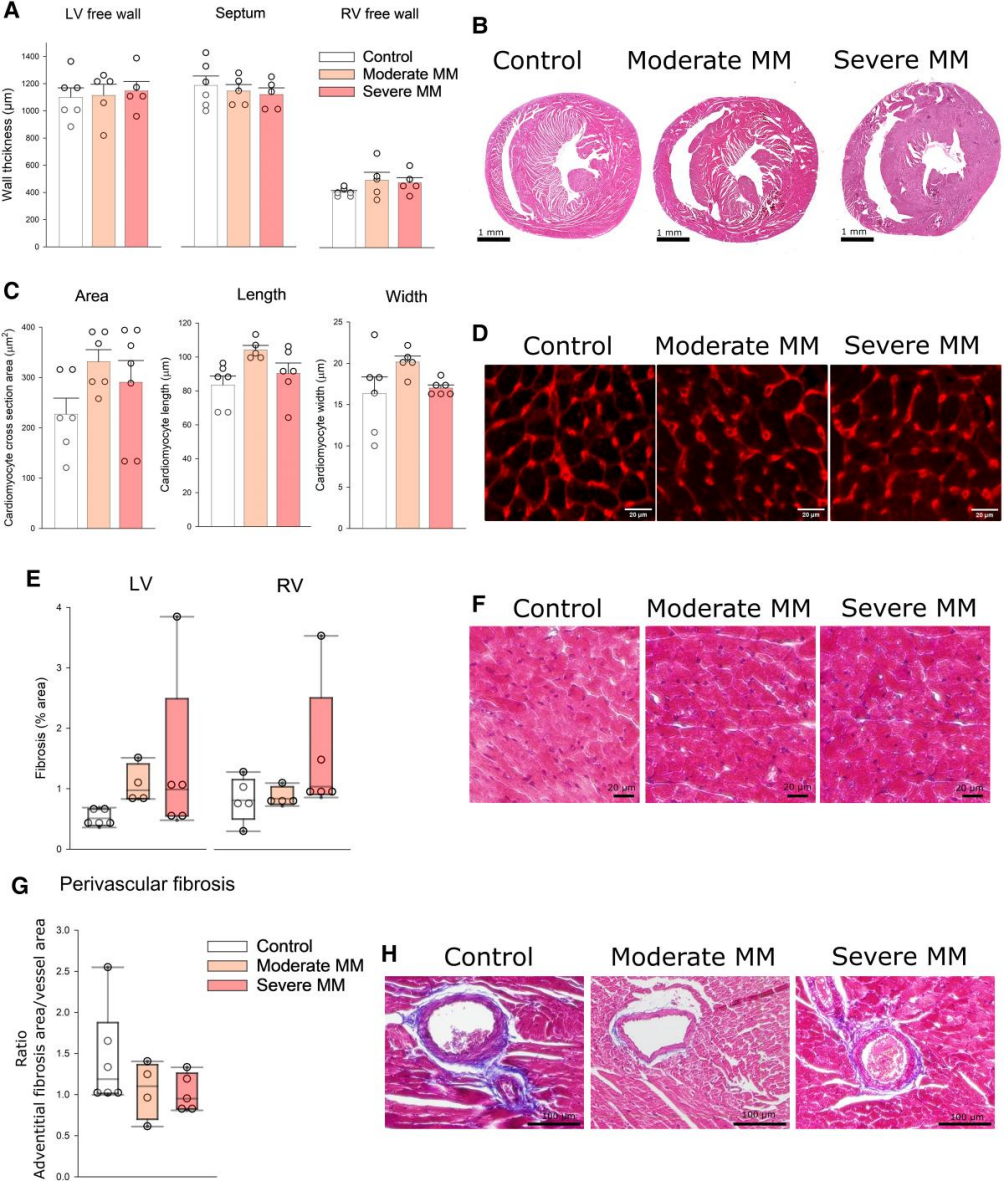
Cardiac histology in Vĸ*MYC MM and control mice. (*A*) LV free wall, septum, and RV free wall thickness obtained from histological images in control, moderate MM, and severe MM mice. Individual dots represent values for a single animal; bars represent means ± SDs. One-way ANOVA did not show any significant differences. (*B*) Representative haematoxylin and eosin cross-section images of mouse hearts from control, moderate MM, and severe MM mice. (*C*) Cardiomyocyte area, length, and width from control, moderate MM, and severe MM hearts. One-way ANOVA did not show any significant differences. (*D*) WGA staining of cardiomyocyte borders: representative images of control, moderate MM, and severe MM hearts. (*E*) Per cent fibrosis in the LV and RV myocardium from control, moderate MM, and severe MM hearts. Circles represent means for individual animals; box plot represents median and interquartile ranges + maximum/minimum. ANOVA on ranks did not show any significant differences. (*F*) Masson’s trichrome staining for collagen: representative images of control, moderate MM, and severe MM hearts. (*G*) Per cent perivascular fibrosis in the LV myocardium from control, moderate MM, and severe MM hearts. ANOVA on ranks did not show any significant differences. (*H*) Masson’s trichrome staining for collagen: representative images of control, moderate MM, and severe MM hearts.

Cardiac histology did not reveal any structural abnormalities in MM hearts: no disturbances of myocardial structure or inflammatory infiltrates, LV, septum, and RV wall thickness did not change (*Figure 5A* and *B*). Also, no significant changes were observed in individual cardiomyocyte size (*Figure 5C* and *D*), confirming lack of myocardial hypertrophy, and no increase of total (*Figure 5E* and *F*) or perivascular (*Figure 5G* and *H*) fibrosis was found, indicating functional rather than structural origin of cardiac dysfunction associated with MM in this mouse model.

## 4. Discussion

Here, we show that MM causes impairment of coronary blood flow and coronary reserve at the capillary level, due to increased blood viscosity secondary to high abnormal monoclonal protein concentration. The impairment exacerbates with the disease progression and is accompanied by abnormalities in cardiac systolic function. This is a unique situation, since in almost every other instance of myocardial ischaemia it is caused by abnormal vascular structure (stenosis of a large epicardial artery caused by an atherosclerotic plaque) or function (abnormal vasodilation of small coronary arterioles causing microvascular angina) that would eventually require completely novel therapeutic approach to prevent cardiac ischaemia.

In the normal coronary circulation, under resting conditions, a vast majority (66%) of coronary resistance resides in the microvasculature, mainly in the small arterioles < 200 µm in diameter (41%),^20^ while the remaining 25% is provided by capillaries. Large arteries provide virtually no resistance and venous resistance is also negligible. Under metabolic stress or with pharmacological challenge (such as adenosine), arteriolar smooth muscles relax, resulting in arteriolar dilation and reduction of coronary resistance and proportional increase of CF. Since capillaries are composed of endothelial cells and lack smooth muscles, they do not dilate and under metabolic stress or adenosine challenge their contribution to coronary resistance increases from 25% to >75%^21^ and they become the dominant player, determining final coronary resistance and coronary flow.

Blood flow through a network of vessels is inversely proportional to its resistance. According to Poiseuille’s law, resistance to flow of a liquid through a tube depends inversely on the radius of the tube to the fourth power and directly on the viscosity of the liquid. While the magnitude of the effect of the radius on vessel resistance does not change with the size of the vessel, this is not the case with viscosity. The effective viscosity actually decreases with the vessel diameter down to approximately 30 µm (so called Fahraeus–Lindqvist effect),^22^ but abruptly increases with further reduction of vessel size, reaching values up to eight-fold higher than predicted in capillaries ∼5 µm in diameter. Therefore, increased blood viscosity disproportionally affects capillary resistance. Postulated mechanism involves interaction between blood components and the vessel wall.^23^ Indeed, variations of blood viscosity were shown to affect predominantly microvascular resistance, while their effect on epicardial coronary resistance and resistance at the stenotic site located in epicardial coronary arteries was negligible.^24^

Under normal conditions, blood viscosity is determined mainly by haematocrit and the effects of plasma proteins are largely negligible.^25^ However, in MM, the abnormal immunoglobulins may constitute the majority of plasma proteins. Of note, there is a large variation in the type of secreted monoclonal proteins (whole immunoglobulins vs. light chains; IgA, IgG, or IgM), although IgG type is the most common form of MM. Here, we show that IgG produced by MM cells in our murine Vĸ*MYC model of MM caused much higher increase of blood and plasma viscosity than predicted by protein concentration alone (*Figure 1D*). Available clinical data indicate that indeed blood viscosity in patients with IgG and IgA MM^26^ or polyclonal hypergammaglobulinemia^27^ is disproportionally high, presumably due to the tendency of IgG to form aggregates in vivo that further increase blood viscosity.^28^

Using intravital microscopy and single RBC tracking in coronary capillaries, we showed that moderate MM reduced CFR, but not the resting CF, while severe MM reduced CF and virtually abolished CFR. Reduction of RBC flow velocity in coronary arterioles in moderate and especially severe MM may seem strange, but it actually is a proof for capillaries providing dominant resistance to blood flow and limiting CF increase under these conditions. Namely, arteriolar dilation accompanied by reduction of RBC flow velocity through arterioles indicates that it was not the arteriolar resistance that limited CF increase, but rather some downstream mechanisms. Similar effects were obtained with a lipid emulsion, Clinoleic, that analogously increased blood viscosity,^29^ providing further support for the hypothesis. Moreover, we did not find any differences in the density or patency of coronary capillaries between control or MM hearts. Lack of differences in coronary resistance between control and MM hearts perfused *ex vivo* with saline supported our in vivo findings, confirming that abnormalities of CF and CFR are indeed caused by some blood related factors rather than vascular abnormalities.

Of note, neither hepatic nor intestinal capillary blood flow was impaired by MM. Both these vascular beds are characterized by low resting capillary resistance: in the intestines due to relatively high venous pressure and venous resistance,^30^ while in the liver due to the fact that 3/4 or hepatic blood flow is provided by the low pressure portal vein system.^31^ This data confirms that hyperviscosity particularly affects the coronary circulation, where a large portion of resting resistance resides in capillaries, more than in low capillary resistance areas, such as the gastrointestinal system.

Impaired arteriolar dilation in moderate and especially severe MM is an interesting finding. It could be related either to improved basal NO release that presumably has already partially dilated arterioles, as was shown for example in the arterioles of hamster skeletal muscle,^32^ exhausting some of their dilatory potential at rest or could be a sign of activation of metabolic mechanisms secondary to ischaemia caused by impaired capillary blood flow (as in conventional stenotic CAD). Combination of these mechanisms is also probable.

To further examine potential effects of MM on the vasculature, we investigated endothelial function using in vivo imaging with DAF-2 DA, a cell permeable fluorescent probe for reactive oxygen species and NO.^33^ DAF-2 DA is membrane permeable and diffuses freely into the cells driven by concentration gradient and is then hydrolysed by cytosolic esterase to form DAF-2. Intracellular DAF-2 is less membrane permeable due to its polarity and is designed to be trapped inside the cells. Since in our experiments we injected the dye into the blood in the LV, only the luminal side of the blood vessels, i.e. endothelial cells, was exposed to DAF-2 DA, minimizing contribution of other cells to the observed NO signal. Here, we showed that baseline NO production was actually increased in the coronary endothelium in MM mice, especially in capillaries and small vessels. This is both consistent with increased shear stress due to increased blood viscosity, especially in vessels < 30 µm in diameter as well as with our previous data obtained in the same MM model using complimentary techniques: Langendorff-perfused hearts and stimulation of endothelium with acetylcholine and bradykinin.^13^ However, plasma concentration of NO metabolites, reflecting systemic NO production, was unchanged, suggesting that this effect could be confined only to specific vascular beds, such as the coronary circulation.

Last but not least, we found impairment of systolic, but not diastolic LV and RV cardiac function both in moderate and severe MM mice. We showed lack of any structural changes in the MM hearts: no abnormalities of gross myocardial structure, hypertrophy at the organ or cellular level, or increased fibrosis that could be responsible for the observed impairment of systolic function. If an ongoing ischaemia was driving the impairment of cardiac function, we would expect primarily diastolic rather than systolic dysfunction, which was not the case here.^34^ We can speculate that repeated bouts of demand-induced ischaemia related to impaired CFR were responsible for abnormal cardiac function in our study. Indeed, both animal^35^ and human^36^ data indicate that cumulative impairment of systolic cardiac function can result from myocardial stunning induced by increased myocardial demand when CFR is reduced. Although completely speculative, this is compatible with our data, since systolic impairment is already present in moderate MM, when resting CF is preserved. Of note, the functional abnormalities were more pronounced and appeared earlier in the LV than in RV, which is consistent with higher resistance to ischaemia/hypoxia of RV vs. LV.^37^ A strong positive correlation between increased blood viscosity and coronary artery disease has also been reported.^38^ There is a case report of a MM patient who had highly increased plasma IgM, signs and symptoms of myocardial ischaemia and impaired CFR. After chemotherapy, her IgM were reduced, signs and symptoms resolved, and CFR increased, supporting our concept of CFR impairment by hyperviscosity.^39^

In summary, we present a new mechanism of CF and CFR impairment associated with MM, at the capillary level. Since capillaries cannot be directly targeted and dilated, other approaches to prevention and treatment of cardiac impairment induced by CF abnormalities in MM are needed, such as metabolic interventions or agents improving blood rheology to reduce blood viscosity.

### 4.1 Limitations

Use of a single model of MM in our study to draw general conclusions regarding cardiac effects of MM is a limitation in view of extremely high variability of human MM. Moreover, due to quite rapid MM progression in our model, we investigated rather acute effects of MM on CF, which may differ from chronic effects encountered in humans with a history of several years of the disease progression. Another limitation is the fact that imaging of RBC flow velocity was confined to subepicardial region of LV due to technical limitations (maximum depth of imaging using our intravital microscope was approximately 50–60 µm), so subendocardial blood vessels were inaccessible using our method.

## Supplementary Material

cvaf164_Supplementary_Data

## Data Availability

Data available on request.
