# Supplementary material for: Impaired coronary flow reserve by hyperviscosity in a mouse model of non-light chain multiple myeloma: a mechanism of coronary flow impairment at the capillary level
Source: Cardiovasc Res. 2025 Sep 16;121(13):2070–81. doi: 10.1093/cvr/cvaf164 (PMC12560772; doi:10.1093/cvr/cvaf164)
Supplement: cvaf164_Supplementary_Data [file cvaf164_supplementary_data.zip › Multiple myeloma - supplementary data.docx]

**Supplementary Figures**

**Supplementary Fig. 1** **Coronary flow and coronary flow reserve in Vĸ*MYC multiple myeloma and control mice – distribution of individual measurements.**


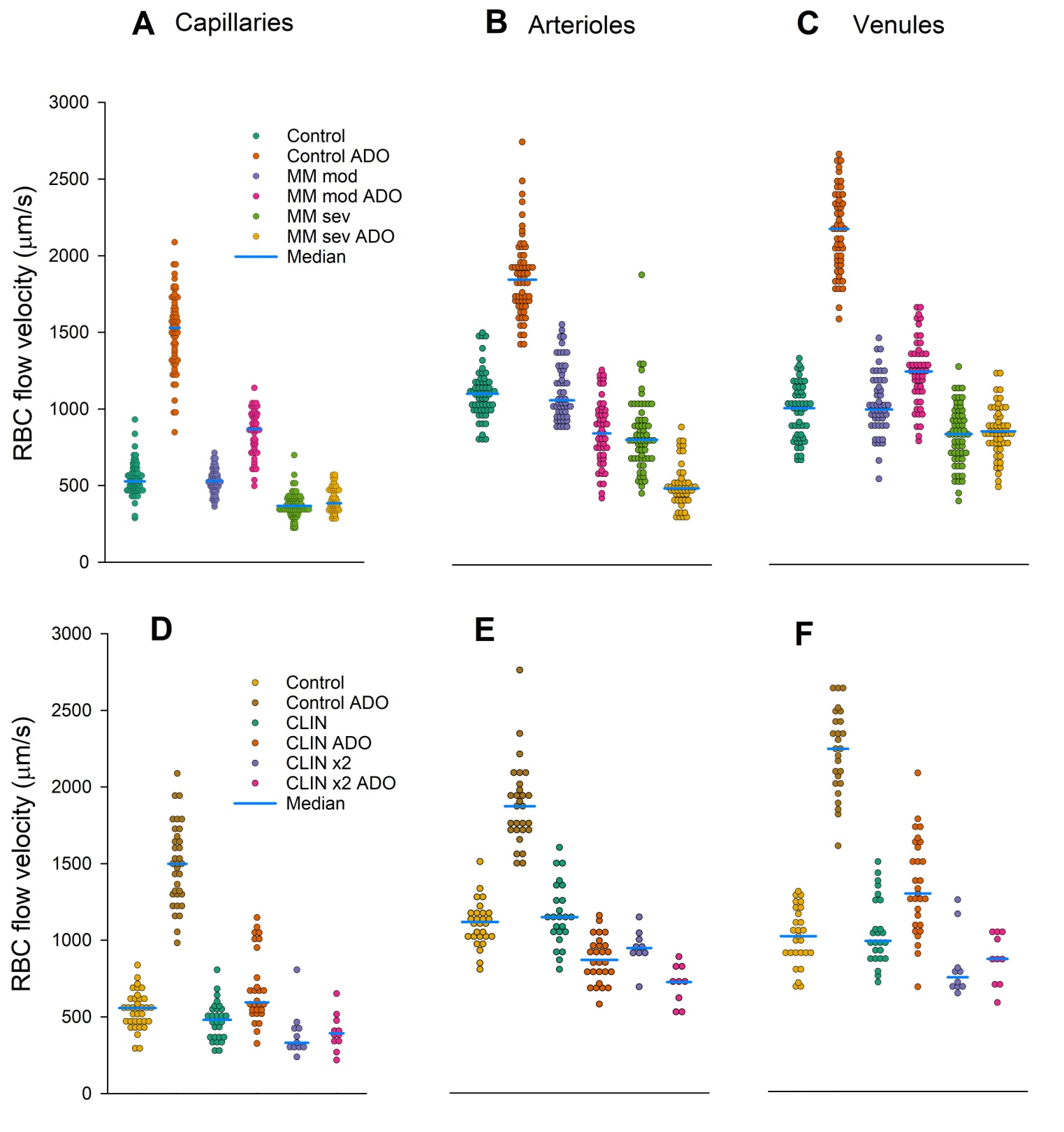


Red blood cell flow velocity in coronary capillaries (A and D), arterioles (B and E) and venules (C and F) in control (A-C n=7, D-F n=5), moderate multiple myeloma (A-C, MM, n=5), severe MM (A-C, n=5) mice and in control mice injected with a lipid emulsion (Clinoleic, CLIN, n=5, 3 µl/g of body weight) and with a double dose of Clinoleic (CLINx2, n=1, 6 µl/g of body weight), at baseline and following injection of a bolus of adenosine (ADO) into the left ventricular chamber. Dots represent individual measurements (technical replicates). No statistical test was performed on this data since it was used to present data distribution.

**Supplementary Fig. 2** **Coronary flow and coronary flow reserve in Vĸ*MYC multiple myeloma and control mice – distribution of individual measurements.**


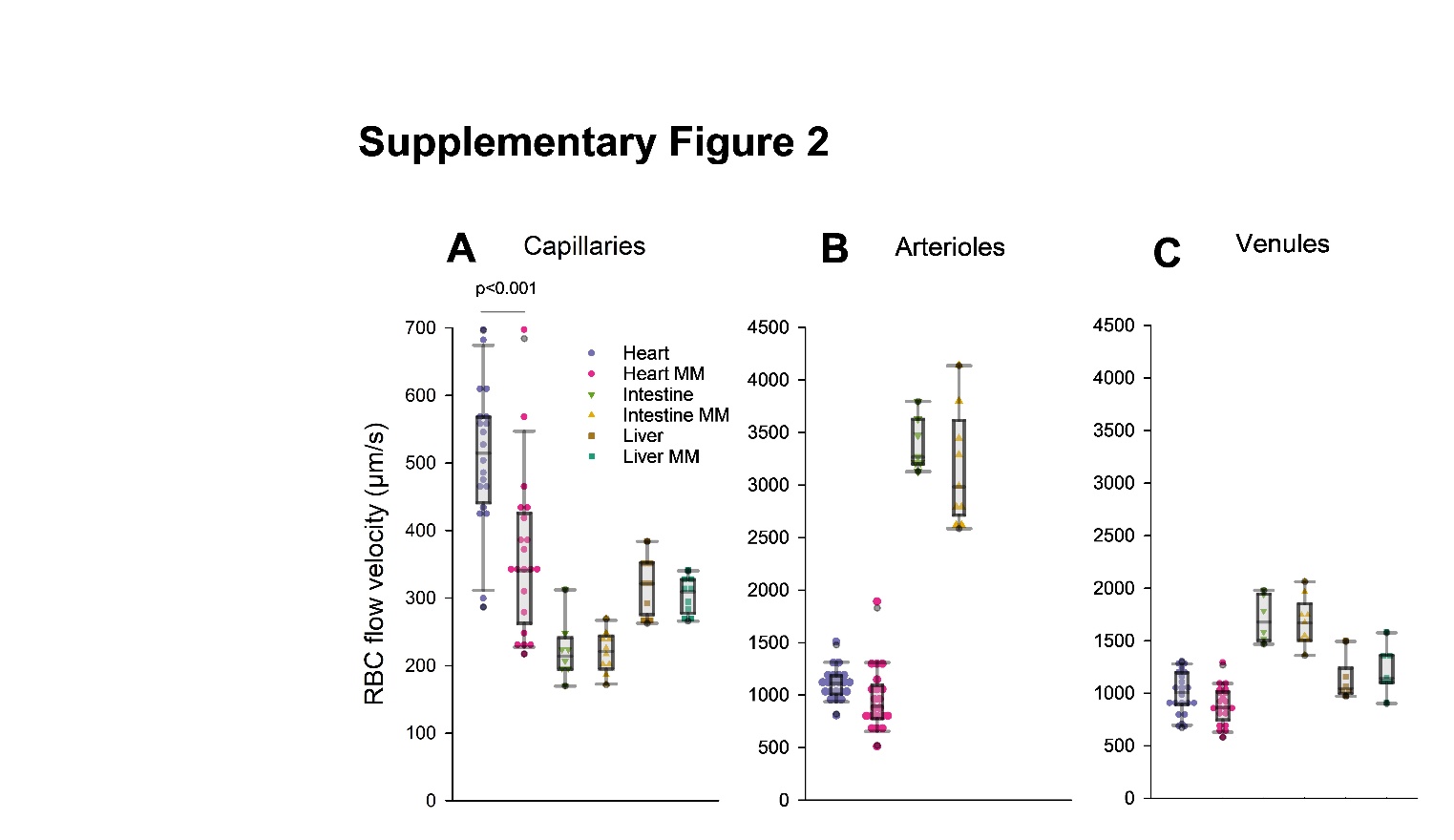


Red blood cell flow velocity in capillaries (A), arterioles (B) and venules (C) of the heart, small intestine and liver in control mice (n=2) and in mice with severe multiple myeloma (MM, n=2). No results were shown for liver arterioles, since we were unable to image them using our intravital microscope. Dots represent individual measurements (technical replicates). Student t-test was used to test differences between control and MM mice.

**Supplementary movies**

**Supplementary movie 1.** A mouse on a ventilator with the hearts exposed and suction chamber adhering to the beating heart; the epicardial blood vessels can be seen.

**Supplementary movie 2.** Stained red blood cells (red) moving through the epicardial capillaries in a control mouse with blood vessels stained blue using anti-CD31 antibody.

**Supplementary movie 3.** Stained red blood cells (red) moving through the epicardial capillaries and a small arteriole in a severe multiple myeloma mouse with blood vessels stained blue using anti-CD31 antibody.

**Supplementary movie 4.** Stained red blood cells (red) moving through the epicardial arteriole in a control mouse with blood vessels stained blue using anti-CD31 antibody.

**Supplementary movie 5.** Stained red blood cells (red) moving through the intestinal small arteriole (from left to right), small intestinal venule (from right to left) and capillaries of intestinal villi in a control mouse with blood stained blue using Evans Blue.
